# Random Survival Forests for Predicting the Bed Occupancy in the Intensive Care Unit

**DOI:** 10.1155/2016/7087053

**Published:** 2016-10-13

**Authors:** Joeri Ruyssinck, Joachim van der Herten, Rein Houthooft, Femke Ongenae, Ivo Couckuyt, Bram Gadeyne, Kirsten Colpaert, Johan Decruyenaere, Filip De Turck, Tom Dhaene

**Affiliations:** ^1^Ghent University-iMinds, Technologiepark 15, 9052 Gent, Belgium; ^2^UZ Ghent, De Pintelaan 185, 9050 Gent, Belgium

## Abstract

Predicting the bed occupancy of an intensive care unit (ICU) is a daunting task. The uncertainty associated with the prognosis of critically ill patients and the random arrival of new patients can lead to capacity problems and the need for reactive measures. In this paper, we work towards a predictive model based on Random Survival Forests which can assist physicians in estimating the bed occupancy. As input data, we make use of the Sequential Organ Failure Assessment (SOFA) score collected and calculated from 4098 patients at two ICU units of Ghent University Hospital over a time period of four years. We compare the performance of our system with a baseline performance and a standard Random Forest regression approach. Our results indicate that Random Survival Forests can effectively be used to assist in the occupancy prediction problem. Furthermore, we show that a group based approach, such as Random Survival Forests, performs better compared to a setting in which the length of stay of a patient is individually assessed.

## 1. Introduction

In recent years, there is an increasing trend to automatically monitor, collect, process, and store clinical parameters of patients during their hospital visit. These automated systems have led to the creation of a vast array of heterogeneous and often incomplete data collections which are hypothesized to contain a wealth of hidden knowledge. However, these data compendia, often dubbed “big data goldmines” in popular media, have mainly been left untouched due to the inherent difficulty they present to (manually) extract information. In this paper, we present our work to create a system which can assist physicians to predict the bed occupancy of an intensive care unit (ICU) based on automatically monitored clinical parameters.

Predicting the amount of free beds in an ICU is a difficult task as there is a substantial amount of uncertainty associated with the prognosis of critically ill patients. This prediction is further complicated by the fact that there is constant arrival of new patients that unexpectedly require intensive care [1]. Nowadays, ICU physicians generally plan only a single day ahead based on clinical judgement whether or not a patient will leave the ICU. This can lead to situations in which capacity problems arise and planned surgeries have to be postponed. The development of an automated system which can assist physicians in these matters would clearly be beneficial to plan better and further ahead. This in turn could help reduce the financial cost associated with an intensive care unit. The latter impact should not be underestimated, as it was reported that the cost of care in 2005 for critically ill patients accounts for about 0.66% of the gross domestic product in the United States [2].

In this work, we use machine learning techniques that are trained with recorded Sequential Organ Failure Assessment (SOFA) scores [3] in order to estimate bed occupancy given the current population of ICU patients. The SOFA score is an established ICU scoring system which assesses the individual degree of organ failure in six organ systems on a daily basis. ICU scoring systems are often automatically gathered and presented at a regular interval to provide physicians a condensed overview of the status and evolution of a patient. Other frequently used scoring systems include, amongst others, APACHE II [4] and SAPS II [5]. A strong connection has been reported between the evolution of the SOFA score and the mortality of patients in ICU wards [6].

In related work, significant efforts have been made to create computational models which predict either the precise length of stay (regression models) or the risk of a prolonged stay (classification algorithms) of patients in the ICU using various clinical parameters. Kramer and Zimmerman [7] have developed methods to identify patients with an increased risk for a prolonged stay at the ICU based on data collected during the first five days of admission. Meyfroidt et al. [8] applied Gaussian processes to predict the length of stay of 960 patients undergoing cardiac surgery using data monitored during the first four hours of admission. Similarly, several studies have used Artificial Neural Networks to predict the length of stay of cardiac patients in different settings [9–11].

Houthooft et al. [12] applied machine learning techniques on SOFA score data to predict individual patient mortality, length of stay, and prolonged stay in the ICU. They conclude that the individual length of stay of a patient is hard to predict and propose a split of patients in a two-by-two grid, based on the mortality risk of the patient and the probability of a prolonged stay. Verburg et al. [13] performed a large comparison of regression models to predict the length of stay of unplanned ICU admissions; they also conclude that it is difficult to predict using only patient characteristics at admission time.

In contrast to these studies, we will not focus on predicting the individual length of stay of a patient but develop a system that predicts the evolution over time of the bed occupancy of an entire ICU given its current population. By opting to model and predict a group of patients instead of predicting at the individual level, we aim to reduce the variability and improve the total accuracy prediction of bed occupancy predictive models. For this, we link the bed occupancy problem to the domain of survival analysis and propose a predictive model using Random Survival Forests [14]. We compare our approach to a standard regression approach in which Random Forests [15] are used to predict the length of stay of each patient.

Note that, in order to predict the exact bed occupancy at time *t*, the arrival of new patients should also be forecasted. However, without actions by physicians (e.g., postpone surgeries), the arrival rate at the ICU is not affected by the number of occupied beds. An estimation of the evolution of available beds based on the current population of patients is required to anticipate problems and act to control new admissions by either reserving additional capacity or moving patients to other hospitals or other wards. These actions are triggered by the ability to detect capacity problems early given the number of planned surgeries, the average interarrival time of critically ill patients, and the forecasted number of occupied beds by the current patients. The approach described in this article aims to deliver a better estimate of the latter aspect.

The article is organised as follows: Section 2 discusses the collection of the data, the scoring system (SOFA), and the applied methods from machine learning. In Section 3, these methods are compared and the results are further analysed.

## 2. Materials and Methods

In this section, we first discuss the properties of the dataset: how it was collected and how the data was further processed. Next, we proceed by introducing the algorithms we use in our work. Finally, we discuss the evaluation metric.

### 2.1. Data Extraction

The data concerns all adult patients admitted between 1 January 2009 and 17 September 2013 at two ICU units of Ghent University Hospital. In total 14,480 patient records were extracted including both monitored values and patient information as well as lab results. ICU stays of over 16 days were rare and did not occur often in our data; hence these patients were excluded as they are more the focus for models that attempt to predict prolonged stays. Furthermore, a large group of patients stays in the ICU for a very short time span. Such patients, with a length of stay of less than 3 days, were also omitted for two reasons. The first reason is that these patients are often recovering from uneventful surgeries and are in good condition, allowing the physician to make an accurate prediction for a short length of stay. The second reason is that the computational model we describe further will use data gathered from the previous three days to make a prediction; therefore the model would need to operate with insufficient data. In addition, ICU planning is mostly concerned with the remaining length of stay for patients with a longer stay. Note that our choice to predict based on 3 days of admission data is short in comparison to other studies (Kramer and Zimmerman [7] and Houthooft et al. [12]). Finally, only the clinical parameters related to the SOFA scoring system were retained. In total, this leads to 4098 unique patient records.

### 2.2. Dataset Processing: SOFA Score Calculations

The SOFA score (Sequential Organ Failure Assessment score) [3] is one of several ICU scoring systems and tries to capture the status of a patient's organ function. The score is in general used to determine the patient's status and evolution throughout his ICU stay and is calculated at a daily interval. The total SOFA score is the sum of six SOFA subscores which are indicators of the coagulation, renal function, cardiovascular system function, respiratory function, liver function, and central nervous system function. For each of the subsystems, a score between 0 and 4 is awarded, with a high score being indicative for organ failure. SOFA scores were automatically calculated by an automated system at the hospital each day at 5 AM using all available clinical parameters up to 24 hours upfront.

The coagulation subscore is calculated by measuring the minimum number of thrombocytes (number of platelets × 10^3^/*μ*L) in the blood of the patient (*α*_coag_) which is then mapped to a SOFA subscore:

(1) $$\begin{array}{c} {SOFA}_{coag}=\left\{\begin{array}{cc} 4 & \alpha_{coag}\leq 20\\ 3 & \alpha_{coag}\leq 50\\ 2 & \alpha_{coag}\leq 100\\ 1 & \alpha_{coag}\leq 150\\ 0 & \alpha_{coag}>150. \end{array}\right. \end{array}$$

The renal function SOFA score is based on two input values: the maximum amount of plasma creatine measured [mg/dL] (*α*_ren_) and the sum of the urine volume [mL] (*β*_ren_):

(2) $$\begin{array}{c} {SOFA}_{renal}=\left\{\begin{array}{cc} 0 & \alpha_{ren}<1.2\\ 1 & \alpha_{ren}<1.9\\ 2 & \alpha_{ren}<3.4\\ 3 & \alpha_{ren}<4.9\vee \beta_{ren}<500\\ 4 & \alpha_{ren}\geq 4.9\vee \beta_{ren}<200. \end{array}\right. \end{array}$$

The liver subscore is calculated by measuring the maximum bilirubin serum value [mg/dL] (*α*_liver_) within the 24 h window:

(3) $$\begin{array}{c} {SOFA}_{liver}=\left\{\begin{array}{cc} 4 & \alpha_{liver}\geq 12\\ 3 & \alpha_{liver}\geq 6\\ 2 & \alpha_{liver}\geq 2\\ 1 & \alpha_{liver}\geq 1.2\\ 0 & \alpha_{liver}<1.2. \end{array}\right. \end{array}$$

To calculate the nervous system subscore, the minimum value of the Glasgow coma score (*α*_cns_) is used. In case the patient was sedated in the last 24 hours, the last known value of the Glasgow coma scale is used:

(4) $$\begin{array}{c} {SOFA}_{cns}=\left\{\begin{array}{cc} 4 & \alpha_{cns}\leq 6\\ 3 & \alpha_{cns}\leq 9\\ 2 & \alpha_{cns}\leq 12\\ 1 & \alpha_{cns}\leq 14\\ 0 & \alpha_{cns}>14. \end{array}\right. \end{array}$$

The respiratory function SOFA score is calculated by measuring the minimum PaO_2_/FiO_2_ ratio (PF) [mm Hg] (*α*_resp_) and by checking whether or not the patient was ventilated (*V*) in the last 24 h period:

(5) $$\begin{array}{c} {SOFA}_{resp}=\left\{\begin{array}{cc} 4 & \alpha_{resp}\leq 100\wedge V\\ 3 & \alpha_{resp}\leq 200\wedge V\\ 2 & \alpha_{resp}\leq 300\\ 1 & \alpha_{resp}\leq 400\\ 0 & \alpha_{resp}>400. \end{array}\right. \end{array}$$

To calculate the cardiovascular system function SOFA score, first the mean arterial pressure (map) (*α*_cardio_) is calculated. Also, the maximum amount of administered doses of the following drugs is extracted in [*μ*g/kg/min]: dopamine (dop), dobutamine (dobu), epinephrine (epi), and norepinephrine (nor):

(6) SOFAcardio=4dop>15∨epi>0.1∨nor>0.13dop∈5,15∨epi∈0,0.1∨nor∈0,0.12dop∈0,5∨dobu>01map<700otherwise.

### 2.3. Dataset Definition

A dataset was created using the processed data, in which the remaining length of stay of a patient at day *d* of his or her stay, measured in days, is defined as the target variable. Each patient in the processed data is split into one or more dataset entries. The predictor values of a single entry are as follows:

(i) The SOFA values of the patient during the previous 3 days (6 × 3 features)
(ii) The amount of days the patient has been admitted to the ICU (1 feature)

For each patient a dataset entry is created at the start of day 4 and at the start of each following day until the morning of discharge. This approach can be regarded as a sliding window over the stay of each patient, creating an entry for each step of the window. The additional parameters summarizing the amount of days the patient has already been admitted encode important information on the status of the patient together with the SOFA scores.

The final dataset contains 19 predictor variables and 11662 entries, generated from 4023 patients. Missing values were imputed as zero because this indicates that according to the doctor's judgement the organ was healthy and no monitoring was necessary.

### 2.4. Random Forests

The Random Forests (RF) is an ensemble learning technique used frequently for supervised learning problems (regression and classification). It consists of an ensemble of decision trees trained on a training set of labeled data points. After training a Random Forest, an unseen data point is predicted as the mode of the output values of the trees (classification) or the mean of the predictions of the trees (regression).

The algorithm constructs the forest as follows: training is initiated by drawing *B* bootstrap samples from the original dataset: for each bootstrap the excluded data is referred to as Out-of-Bag (OOB) data. Next, a decision tree for each bootstrap sample is grown: the root node is split into two daughter nodes. These daughter nodes are recursively split until no new daughter nodes can be formed because those would no longer hold more than a predefined minimum number of data points or when the tree exceeds the maximum depth specified. These extreme nodes which can no longer be split are referred to as* terminal nodes*. The terminal nodes of a decision tree *b* are denoted by the set *T*(*b*). To split a node, a random set of the candidate variables *p* is chosen, and an optimal split value *c* is calculated by optimizing an information metric. Popular metrics are the Gini impurity, the information gain, variance reduction, or the residual sum of squares. The latter metric is used for the experiments in this work. A more in-depth overview is given in [16].

For regression, each terminal node *h* of a tree *b* has an associated value *F*(*h*). In case *h* contains only a single training data point, the value corresponds to the value of the training point. If several training points are associated with the node, the average is taken. To predict an unseen data point **x** with tree *b*, the point is first dropped through the tree until it ends in a terminal node *h*. The prediction of a tree node can be written as

(7) $$\begin{array}{c} H_{b,h}\left(\;x_{i}\;\right)=\left\{\begin{array}{cc} F\left(h\right) & x_{i}\in h\\ 0 & \text{otherwise}; \end{array}\right. \end{array}$$

the predicted output of the RF ensemble of trees can then be written as

(8) $$\begin{array}{c} H_{e}\left(\;t,x_{i}\;\right)=\frac{1}{B}\overset{B}{\underset{b=1\;}{\sum }}\underset{h\in T\left(b\right)}{\sum }H_{b,h}\left(\;x_{i}\;\right). \end{array}$$

Note that dropping **x**_*i*_ through a decision tree ends in a single terminal node due to its binary nature.

### 2.5. Baseline Approach

A second method to predict the LOS of a patient is also included in Results and Discussion. In this baseline method, the predicted LOS for a patient **x** is obtained by sampling a random patient from the data and selecting their LOS value. As the random numbers are drawn from the data distribution rather than a different distribution, this provides a competitive baseline which acts as a reference for the results obtained with RSF and RF.

### 2.6. Survival Analysis and Random Survival Forests

Survival analysis concerns a class of statistical methods for analysing “time to event” data which occurs in a number of research fields such as medicine, biology, and engineering. An event can, for example, be a failure of a component in engineering or, in the case of treatment of ill patients, death. A typical feature of this type of data is that it often contains censored or truncated data observations. For example, right censored data points are observations, where all available information is that the event did not occur yet at a given time. Random Survival Forests (RSF) were introduced by [14] as a forest ensemble learner for the analysis of right censored data. The length of stay of a patient at the ICU can be considered as an unusual instance of censoring, in which entering the ICU is considered to be a “birth” event, leaving the ICU a “death” event and the death of an individual is considered to be right censoring.

The RSF algorithm initiates similar to RF by drawing *B* bootstrap samples from the original dataset. For each bootstrap sample, a survival tree is grown: nodes are recursively split until no new daughter nodes can be formed because those would no longer hold more than *d*_0_ > 0 unique death events. Good splits maximize the survival difference between the daughters nodes; in the experiments this was determined using the log-rank splitting rule [17]. The values in the terminal nodes are given by a Cumulative Hazard Function and are time-dependent. For a terminal node *h* of a survival tree *b* at time *t*, this is given by the Nelson-Aalen estimator:

(9) $$\begin{array}{c} N_{b,h}\left(\;t\;\right)=\underset{t_{l,h}\leq t}{\sum }\frac{d_{l,h}}{I_{l,h}}, \end{array}$$

which sums the ratio of the number of deaths *d*_*l*,*h*_ and the individuals at risk *I*_*l*,*h*_ over all distinct time events *t*_*l*,*h*_ prior or equal to *t*. All cases of *h* are assigned the same CHF. To compute the ensemble CHF of the survival forest with *B* trees for a given* d*-dimensional case **x**_*i*_,

(10) $$\begin{array}{c} H_{e}^{s}\left(\;t,x_{i}\;\right)=\frac{1}{B}\overset{B}{\underset{b=1\;}{\sum }}\underset{h\in T\left(b\right)}{\sum }H_{b,h}^{s}\left(\;t,x_{i}\;\right), \end{array}$$

with *H*_*b*,*h*_^*s*^(*t*, **x**_*i*_) being equal to the Nelson-Aalen estimator if **x**_*i*_ ends in *h* when dropped through the survival tree:

(11) $$\begin{array}{c} H_{b,h}^{s}\left(\;t,x_{i}\;\right)=\left\{\begin{array}{cc} N_{b,h}\left(t\right) & x_{i}\in h\\ 0 & \text{otherwise.} \end{array}\right. \end{array}$$

It can be observed that the main points of difference with the traditional RF are the values associated with each terminal node. These are given by the Nelson-Aalen estimator and depend on the time *t*.

### 2.7. Using Random Survival Forests to Predict the ICU Bed Occupancy Over Time

In order to predict the bed occupancy of the ICU over time, we use the following approach: define the survival function *S*(*t*) as the probability that a patient will still be admitted at the ICU:

(12) $$\begin{array}{c} S\left(\;t\;\right)=P\left(\;\left\{\;T<t\;\right\}\;\right). \end{array}$$

Note that this survival function is reflected by the Nelson-Aalen estimator as used by RSF. We now approach the bed occupancy problem as follows:

(1) Grow a Random Survival Forest using training data.
(2) For a test set of patients currently admitted at the ICU, drop each patient down the forest.
(3) Extract the individual predicted survival functions for each patient by evaluating the obtained CHFs (see (10)) over a time interval.
(4) Sum these survival functions at each time point and consider the obtained vector, load_pred_, as the expected amount of patients at each time point.

**Figure 1:**
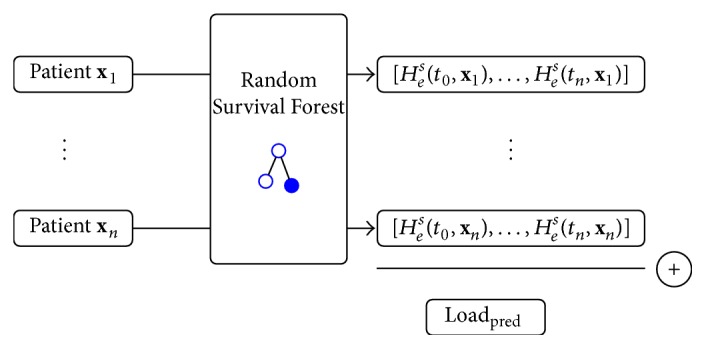
Schematic illustration of the computation of load_pred_, given a set of patient data.

Figure 1 illustrates how load_pred_ is obtained from a set of patient data, using a trained Random Survival Forest (steps (3) and (4)).

### 2.8. Goal and Error Definition

Ultimately, our goal is to develop and assess a computational model which can predict the occupancy of the ICU for the next several days. This model will use the predictor variables corresponding to the patients currently admitted to the ICU. As described in the previous section, these are the SOFA scores of the previous three days and the amount of days the patient has been admitted. As output, the predicted occupancy of the ICU at each day is to be returned, as if no new patients would be admitted.

To assess the accuracy of such a method, standard 10-fold cross validation was used, including a modification to calculate a more intuitive measure, error measure *E*, for practical use. For each test fold, a set of 25 dataset entries is sampled without replacement, of which load_pred_ is predicted using the trained forest. This set can be considered as a random occupation of the ICU with a group of patients with varying length of stay and days admitted. Next, the mean absolute error between load_pred_ and the actual load_real_ is calculated. As a final step, load_pred_ and load_real_ are truncated at day 10, as in practice the prediction accuracy of the models after this time period is less important. Note that the truncation occurs after calculating load_pred_ and load_real_; hence it does not influence the models to predict beyond day 10 if desired. This process is repeated 100 times and averaged to obtain *E*. Using this scheme, *E* can be interpreted intuitively as the average error of the occupancy over time in an average sized ICU of 25 available beds in the first 10 days.

## 3. Results and Discussion

In this section, we first evaluate our approach using the error measure described in the previous section. In addition, we further investigate which clinical parameters the algorithms indicate as important to make the prediction.

### 3.1. Performance Evaluation

We compare our proposed RSF approach with both a standard Random Forest regression (RF) model trained to predict the individual length of stay of a single patient and the baseline approach.

**Figure 2:**
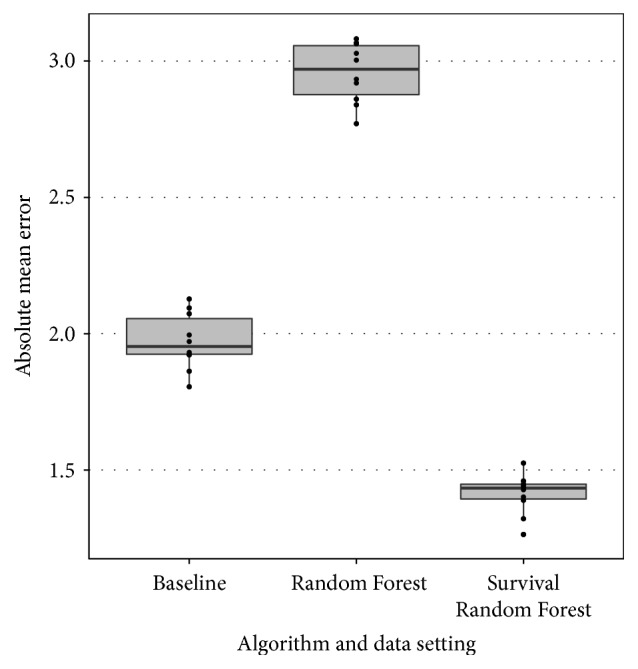
Boxplot for error measure *E* for the three methods considered: baseline (B), Random Forests (R), and Random Survival Forests (S).

Figure 2 plots the error measure *E* for all algorithms in a boxplot overview. Each individual dot in the boxplot represents a single result of *E* in one of the 10 folds. To support the observations in the remainder of this section, we verified their statistical significance by means of the Wilcoxon rank-sum test with continuity correction (cut-off *p* value ≤0.05).

A first observation is that the Random Forest regression approach performs worse than the baseline approach. We believe that this is due to the fact that the RF method is not able to accurately model and predict the individual length of stay of a patient with the given data. Instead, the model resorts to predicting the average length of a stay of a patient in many cases to minimize the prediction error for every individual, which leads to inaccurate predictions at the group level. By focusing on a group based approach that produces a day-by-day estimate by summing individual predictions this problem could be avoided. The RSF method does this, as probabilities are predicted on the individual level for each point in time by the survival functions.

A second observation is that the RSF approach performs significantly better than the baseline approach. This indicates that it is possible to construct machine learning models that use SOFA scores to better predict the bed occupancy. In our opinion, this is a strong result as two random groups of 25 patients in the average case in the same ward should not lead to big differences in bed occupancy over time. Therefore, any method that can significantly improve on the latter is a useful tool in practice.

### 3.2. Contribution of the Variables to the Predictive Power of the Model

**Figure 3:**
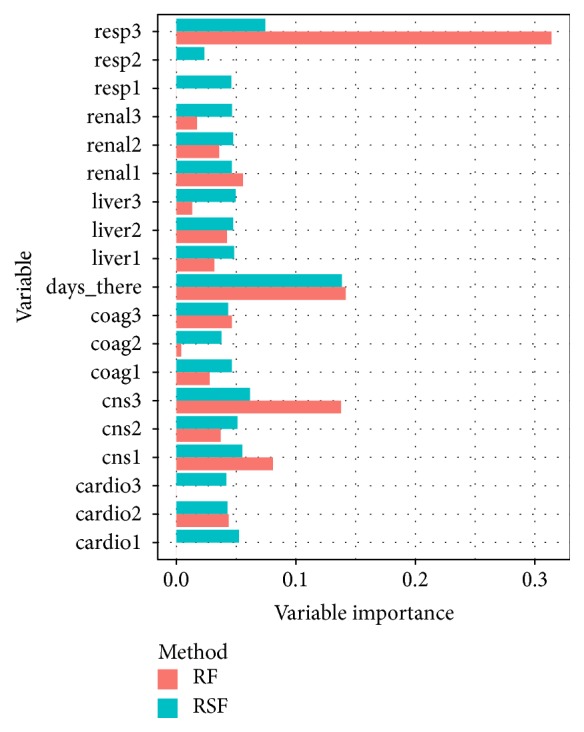
The relative variable importance measure for the Random Forest and Random Survival Forest.

Both Random Forest and Survival Random Forests can provide further insight in which variables, in this case, SOFA scores, are informative to make predictions. In the setting of both Regression Random Forests and Random Survival Forests, the Breiman-Cutler permutation variable importance measure [15] has been proposed. Briefly, this measure is obtained by comparing the normal OOB prediction error to the OOB prediction error when a certain feature *x* is randomly permuted for each tree and this further averaged over all trees. These measures were calculated for both algorithms and were further normalized to obtain relative scores as plotted in Figure 3. A first observation which can be made is that ${SOFA}_{resp}$ measure at day 3 is especially informative in both algorithms. Furthermore, both algorithms also indicate ${SOFA}_{cns}$ at day three as a major second contributor. Both algorithms also indicate that the amount of days the patient has already been admitted is a major predictor for the remaining length of stay. Further down the ranking, the relative importance is more spread across all features. This difference is more pronounced in RSF with some variables, for example, ${SOFA}_{coag}$ not contributing to the predictive power of the model. Next, a clear trend is that the information available at day 3 is generally more informative than the scores at days 2 and 1. This is to be expected, as the latest recorded scores provide the most up to date information on the condition and evolution of the patient.

## 4. Conclusion

Previous studies have concluded that modelling the individual length of stay of patients admitted in the ICU is a nontrivial task. In this work, we have explored and proposed a group based approach to the problem by constructing a novel data-driven algorithm to predict the overall bed occupancy of an ICU. Our approach uses Random Survival Forests to create individual survival functions which are then summed over time to obtain the overall bed occupancy prediction at each day. We have shown that this approach is to be preferred over a standard regression approach and performs significantly better than a baseline approach. Finally, we have also shown that the respiratory and nervous system SOFA scores together with the number of days the patient has already been admitted are the most important measures and that the information of day 3 is the most influential.

## References

[1] Quinn T. D., Gabriel R. A., Dutton R. P., Urman R. D. (2015). Analysis of unplanned postoperative admissions to the intensive care unit. *Journal of Intensive Care Medicine*.

[2] Halpern N. A., Pastores S. M. (2010). Critical care medicine in the United States 2000–2005: an analysis of bed numbers, occupancy rates, payer mix, and costs. *Critical Care Medicine*.

[3] Vincent J.-L., Moreno R., Takala J. (1996). The SOFA (Sepsis-related Organ Failure Assessment) score to describe organ dysfunction/failure. *Intensive Care Medicine*.

[4] Knaus W. A., Draper E. A., Wagner D. P., Zimmerman J. E. (1985). APACHE II: a severity of disease classification system. *Critical Care Medicine*.

[5] Le Gall J.-R., Lemeshow S., Saulnier F. (1993). A new simplified Acute Physiology Score (SAPS II) based on a European/North American multicenter study. *Journal of the American Medical Association*.

[6] Vincent J.-L., Moreno R. (2010). Clinical review: scoring systems in the critically ill. *Critical Care*.

[7] Kramer A. A., Zimmerman J. E. (2010). A predictive model for the early identification of patients at risk for a prolonged intensive care unit length of stay. *BMC Medical Informatics and Decision Making*.

[8] Meyfroidt G., Güiza F., Cottem D. (2011). Computerized prediction of intensive care unit discharge after cardiac surgery: development and validation of a Gaussian processes model. *BMC Medical Informatics and Decision Making*.

[9] LaFaro R. J., Pothula S., Kubal K. P. (2015). Neural network prediction of ICU length of stay following cardiac surgery based on pre-incision variables. *PLoS ONE*.

[10] (Jennifer) Tsai P.-F., Chen P.-C., Chen Y.-Y. (2016). Length of hospital stay prediction at the admission stage for cardiology patients using artificial neural network. *Journal of Healthcare Engineering*.

[11] Rowan M., Ryan T., Hegarty F., O'Hare N. (2007). The use of artificial neural networks to stratify the length of stay of cardiac patients based on preoperative and initial postoperative factors. *Artificial Intelligence in Medicine*.

[12] Houthooft R., Ruyssinck J., van der Herten J. (2015). Predictive modelling of survival and length of stay in critically ill patients using sequential organ failure scores. *Artificial Intelligence in Medicine*.

[13] Verburg I. W. M., de Keizer N. F., de Jonge E., Peek N. (2014). Comparison of regression methods for modeling intensive care length of stay. *PLoS ONE*.

[14] Ishwaran H., Kogalur U. B., Blackstone E., Lauer M. S. (2008). Random survival forests. *The Annals of Applied Statistics*.

[15] Breiman L. (2001). Random forests. *Machine Learning*.

[16] Rokach L., Maimon O. (2005). Top-down induction of decision trees classifiers—a survey. *IEEE Transactions on Systems, Man and Cybernetics Part C: Applications and Reviews*.

[17] LeBlanc M., Crowley J. (1993). Survival trees by goodness of split. *Journal of the American Statistical Association*.

